# Nlrp3 Deficiency Alleviates Angiotensin II-Induced Cardiomyopathy by Inhibiting Mitochondrial Dysfunction

**DOI:** 10.1155/2021/6679100

**Published:** 2021-02-05

**Authors:** Yu Chen, Meiying Zeng, Yang Zhang, Hui Guo, Wei Ding, Ting Sun

**Affiliations:** ^1^Department of Cardiology, Shanghai Ninth People's Hospital, School of Medicine, Shanghai Jiao Tong University, Shanghai 200011, China; ^2^Department of Cardiology, Shanghai Jiao Tong University Affiliated Sixth People's Hospital, Shanghai 200233, China; ^3^Department of Radiology, Shanghai Tenth People's Hospital, Tongji University School of Medicine, Shanghai 200072, China; ^4^Department of Cardiology, Shanghai Deji Hospital, Qingdao University, Shanghai 200331, China; ^5^Department of Nephrology, Shanghai Ninth People's Hospital, School of Medicine, Shanghai Jiao Tong University, Shanghai 200011, China

## Abstract

Inflammation has been considered a key component in the pathogenesis and progression of angiotensin II- (Ang II-) induced cardiac hypertrophy and related cardiomyopathy. As a vital mediator of inflammation, the role of the Nlrp3 inflammasome in Ang II-induced cardiomyopathy remains unclear. This study was aimed to determine whether Nlrp3 inflammasome activation and its downstream pathway were involved in Ang II-induced cardiomyopathy. We established an Ang II infusion model in both wild-type and Nlrp3^−/−^ mice to determine the contribution of Nlrp3 to cardiac function. Cardiac fibrosis was determined by Masson's trichrome staining, real-time PCR, and TUNEL assay; cardiac function was assessed by echocardiography. Nlrp3 inflammasome activation and related downstream cytokines were measured by Western blotting and enzyme-linked immunosorbent assays; mitochondrial dysfunction was examined by transmission electron microscopy and real-time PCR. We found that Ang II-infused mice showed impaired cardiac function, as evidenced by increased cardiac fibrosis, apoptosis, inflammation, and left ventricular dysfunction. However, these alterations were significantly alleviated in the mice with Nlrp3 gene deletion. Moreover, Ang II-infused mice showed increased Nlrp3 inflammasome activity relative to that of the cytokines IL-1*β* and IL-18, increased reactive oxygen species, mitochondrial abnormalities, and decreased mtDNA copy number and ATP synthase activity. These molecular and pathological alterations were also attenuated in Nlrp3 deficient mice. In conclusion, Nlrp3 inflammasome-induced mitochondrial dysfunction is involved in Ang II-induced cardiomyopathy. Nlrp3 gene deletion attenuated mitochondrial abnormalities, cardiac inflammation, oxidative stress, and fibrosis and thus alleviated heart dysfunction and hypertrophy. Targeting the Nlrp3 inflammasome and/or mitochondria may be a therapeutic approach for Ang II-induced cardiac diseases.

## 1. Introduction

Cardiovascular disease (CVD) has a high prevalence and has become a major cause of death worldwide [1]. Cardiac hypertrophy plays a key role in the pathological development of CVD. Excessive intrinsic and extrinsic stimuli, such as inflammation and oxidative stress, can cause cardiac hypertrophy, ultimately leading to cardiomyopathy and heart failure [2]. However, the exact mechanism of cardiac hypertrophy remains unclear. Therefore, exploring the underlying pathway may provide new strategies for the prevention and treatment of CVDs.

The renin-angiotensin system (RAS) plays an important role in the pathogenesis and progression of cardiomyopathy and other related CVDs. Angiotensin II (Ang II) is widely accepted as a powerful vasoconstrictor and proinflammatory effector and contributes to hypertension and cardiac fibrosis [3]. Sadoshima et al. found that Ang II significantly increased vasoconstriction, fibrosis, and activation of the immune response, which eventually led to hypertension and hypertrophy [4]. Dahlöf showed that the pathogenesis of cardiac hypertrophy was related to RAS activation, and excessive production of angiotensin II was believed to be responsible. Treatment with Ang II antagonists significantly reversed left ventricular hypertrophy in hypertensive patients [5]. Inflammation is considered a key component in the development of Ang II-induced cardiac remodeling and cardiomyopathy; the protection of blood pressure by AT1 receptor blockers was partly mediated by inhibition of the Ang II-induced inflammatory response [6]. However, the exact mechanism underlying Ang II-induced inflammation remains unknown. Previous research has shown that Ang II drives cardiac inflammation/fibrosis in the context of cardiac dysfunction via endothelial Nox2, whereas several papers demonstrate Nlrp3 involvement in Ang II and cardiac myopathies [7, 8].

Recently, a growing body of evidence has suggested that the inflammasome is an important mediator of innate immunity. The best-known inflammasome is the nucleotide-binding domain and leucine-rich repeat-containing PYD-3 (Nlrp3) inflammasome, which is a multiprotein complex that includes ASC (apoptosis-associated speck-like protein containing a CARD), procaspase-1, and Nlrp3 and plays a key role in sterile inflammatory reactions. In response to various kinds of danger signals (reactive oxygen species [ROS] or K^+^ efflux), Nlrp3 recruits ASC and procaspase-1, and then, Nlrp3 inflammasome oligomerization promotes autocatalytic activation of procaspase-1 and processing of the mature cytokines IL-1*β* and IL-18 [9]. Sandanger et al. demonstrated that the NLRP3 inflammasome was upregulated in cardiac fibroblasts and mediated myocardial ischemia-reperfusion injury [10]. Bracey and colleagues showed that the Nlrp3 inflammasome promoted myocardial dysfunction in structural cardiomyopathy mediated via IL-1*β* [11]. However, the effects and potential downstream pathway of Nlrp3 inflammasome activation in Ang II-induced cardiomyopathy remain unclear.

In addition to inflammation, mitochondrial dysfunction has recently been considered to be a major cause of and plays a pathogenic role in heart failure [12]. Mitochondria are complex intracellular organelles that are involved in various kinds of metabolic processes, such as signal transduction, ROS production, and energy generation. Under pathological conditions, mitochondrial damage increases ROS generation, reduces ATP production, and causes mitochondrial DNA damage, which in turn exacerbate mitochondrial dysfunction and tissue damage [13]. Our previous study demonstrated that Nlrp3 deficiency attenuated mitochondrial dysfunction and renal fibrosis in a model of unilateral ureteral obstruction (UUO) [14]. However, the role of the Nlrp3 inflammasome in regulating mitochondrial dysfunction in Ang II-induced cardiac hypertrophy and cardiomyopathy remains unknown.

In the present study, we investigated the effects and potential downstream pathway of Nlrp3 inflammasome activation in Ang II-induced cardiomyopathy, and whether targeting the Nlrp3 inflammasome could be a novel therapeutic approach for the treatment of Ang II-induced cardiac diseases.

## 2. Materials and Methods

### 2.1. Animal Study and Experimental Protocol

The study protocol was approved by the Ethics Committee of Shanghai Jiao Tong University. Nlrp3^−/−^ mice on a C57BL/6J background were purchased from the Jackson Laboratory (Sacramento, CA, USA). Male Nlrp3^−/−^ (KO) mice and wild-type (WT) mice aged 10-12 weeks (22-26 g) were treated with Ang II or vehicle. An osmotic minipump (Alzet model 2004) was implanted subcutaneously to infuse Ang II (Ang II 500 ng/kg/min) or vehicle control (0.9% saline) for 4 weeks. Ang II (500 ng/kg/min) was used in our experiment according to previous reports [15, 16]. The mice were allowed to recover with free access to food and drinking water. After 4 weeks, the mice were euthanized; blood samples were collected, and heart tissues were collected and immediately frozen in liquid nitrogen for storage at -80°C.

### 2.2. Histological Analysis

After the mice were anesthetized, their hearts were excised and immediately fixed in 4% paraformaldehyde for histological evaluation. Tissue sections (4-*μ*m thick) were prepared, and the total collagen content in myocardial tissue was measured by staining with Masson's trichrome. The results are shown as the percentage of collagen/total myocardial area according to a previous study [17]. Apoptosis in heart tissue was measured using a TUNEL assay and examined using an In Situ Cell Death Detection Kit (Roche, Netley, NJ, USA) according to the manufacturer's protocol. In brief, the deparaffinized sections were incubated with proteinase K, followed by treatment with 0.3% hydrogen peroxide at room temperature for 30 min. After incubation with the TUNEL reaction mixture in the dark for 60 min, 10 fields were selected randomly for each section, and TUNEL-positive cells were counted and averaged. All histological examinations were performed in a blinded fashion.

### 2.3. Echocardiographic Evaluation

Echocardiography was performed to examine cardiac function after 4 weeks. M-mode echocardiography was performed using a high-resolution imaging system (Vevo 770; Fujifilm VisualSonics, Tokyo, Japan) and a 30-MHz imaging transducer. The mice were anesthetized and maintained at a body temperature of 37°C. The echocardiographic parameters measured were left ventricular fractional shortening (LVFS), left ventricular ejection fraction (LVEF), left ventricular internal dimension systole (LVIDs), and left ventricular internal dimension diastole (LVIDd).

### 2.4. Real-Time PCR

Total RNA was extracted from the left ventricle using TRIzol reagent (Invitrogen, Carlsbad, CA) and reverse-transcribed into cDNA according to the instructions of the PrimeScript RT reagent kit (Takara, Dalian, Liaoning, China). RT-PCR analysis was performed using a 7500 Fast Real-Time PCR System (Applied Biosystems, Rockford, IL, USA) as previously described. Relative mRNA expression was normalized to that of GAPDH and is presented as the fold change in expression compared to the control. The primer sequences used were as follows: mtDNA: 5′-TTTTATCTGCATCTGAGTTTAATCCTGT-3′ (F) and 5′-C-CACTTCATCTTACCATTTATTATCGC-3′ (R); ATP synthase: 5′-TCCATCAAAAAC-ATCCAGAAAA-3′ (F) and 5′-GAGGAGTGAATAGCACCACAAA-3′ (R); 18S: 5′-T-TCG-GAACTGAGGCCATGATT-3′ (F) and 5′-TTTCGCTCTGGTCCGTCTTG-3′ (R); fibronectin: 5′-GCGACGGTATTCTGTAAAGTGG-3′ (F) and 5′-GGACAGGGCTTTGGCAGTT-3′ (R); collagen I: 5′-AGGGTCATCGTGGCTTCTCT-3′ (F) and 5′-CAGGCTCTTGAGGGTAGTGT-3′ (R); and GAPDH: 5′-AGGTCGGTGTGAACGGATTTG-3′ (F) and 5′-TGTAGACCATGTAGTTGAGGTCA-3′ (R).

### 2.5. Western Blot Analysis

Western blotting was performed as described [18]. Total proteins were separated by SDS-PAGE and then blotted to a nitrocellulose membrane. After blotting, the membranes were incubated overnight with antibodies against caspase-3, TFAM (Cell Signaling Technology, Beverly, MA), Nlrp3 (AdipoGen, San Diego, CA), IL-18, PGC1a (Santa Cruz Biotechnology, Santa Cruz, CA), and IL-1*β* (R&D Systems, Minneapolis, MN) (all diluted with Tris-buffered saline-Tween 20 [TBST] containing 5% BSA). After being washed with TBST, the blots were incubated with secondary antibodies for 2 h. Immune complexes were visualized with an enhanced chemiluminescent system (Amersham, Little Chalfont, Bucks., UK), and band intensities were quantified using the Quantity One software (Bio-Rad, Hercules, CA, USA).

### 2.6. Transmission Electron Microscopy

Approximately 1-mm^3^ of tissue was obtained from the left ventricle and then fixed with 2.5% glutaraldehyde at room temperature. To examine the mitochondrial ultrastructural morphology, ultrathin sections were prepared, and then, these sections were placed on copper grids and stained with uranyl acetate and lead citrate for evaluation by transmission electron microscopy (TEM).

### 2.7. 8-OHdG Activity and MDA Measurement

Serum 8-OHdG activity and myocardial MDA levels were determined using commercial kits (Beyotime Institute of Biotechnology). All experimental procedures were performed according to the manufacturer's instructions.

### 2.8. Serum IL-1*β* and IL-18 Assay

Serum IL-1*β* and IL-18 levels were measured with enzyme-linked immunosorbent assay (ELISA) kits (RayBiotech, Norcross, GA) according to the manufacturer's instructions [19, 20].

### 2.9. Statistical Analysis

All data are presented as the means ± SEM. Comparisons among groups were performed using one-way analysis of variance followed by Tukey's post hoc test. A value of *P* < 0.05 was statistically significant.

## 3. Results

### 3.1. Nlrp3 Deletion Attenuated Ang II-Induced Cardiac Fibrosis

**Figure 1:**
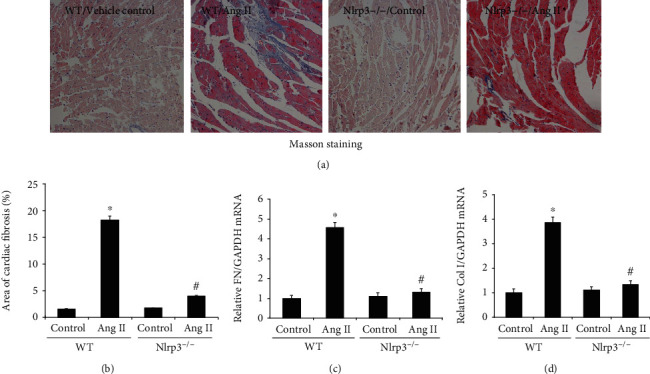
Nlrp3 deletion attenuated Ang II-induced cardiac fibrosis. Representative photomicrographs of Masson's trichrome-stained heart sections ((a) magnification ×400); the area of cardiac fibrosis (%) was evaluated as described in the Materials and Methods section (b). Semiquantitative analysis of heart fibronectin (c) and collagen I (d). mRNA expression was normalized to GAPDH and measured using real-time PCR. All values are means ± SEM (*n* = 6). ^∗^*P* < 0.05 versus the WT/vehicle control group; ^#^*P* < 0.05 versus the WT/Ang II group. FN: fibronectin; Col I: collagen I.

As shown in Figures 1(a) and 1(b), in comparison with the WT/vehicle control group mice, mice in the WT/Ang II group demonstrated significantly increased myocardial collagen levels, as shown by Masson staining. However, the area of cardiac fibrosis was significantly decreased in the NLRP3^−/−^/Ang II group. Similarly, the mRNA levels of fibronectin (4.5-fold) and collagen I (3.8-fold) were markedly elevated in the WT/Ang II group compared with those in the WT/vehicle control group, and Nlrp3 deletion also significantly decreased these mRNA levels relative to those in the NLRP3^−/−^/Ang II group (Figures 1(c) and 1(d)).

### 3.2. Nlrp3 Deletion Decreased Ang II-Induced Myocardial Apoptosis

**Figure 2:**
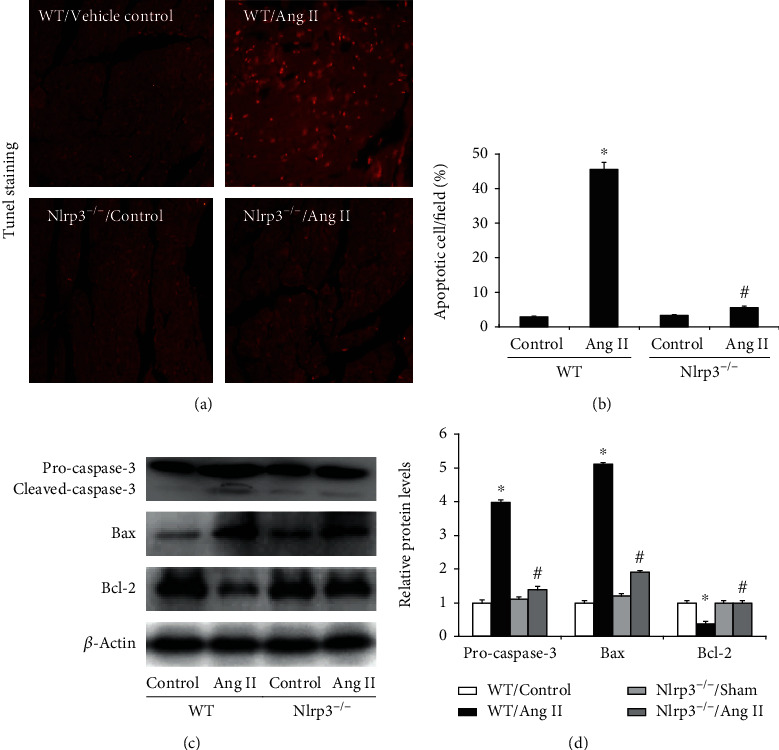
Nlrp3 deletion decreased Ang II-induced myocardial apoptosis. Representative images of TUNEL staining are shown ((a) magnification ×400). The bar graph shows the mean number of TUNEL-positive cardiomyocytes per field (b). The protein expression of caspase 3 was assessed in heart samples (c). Semiquantitative analysis of caspase 3 normalized to *β*-actin (d). All values are the means ± SEM (*n* = 6). ^∗^*P* < 0.05 versus the WT/vehicle control group; ^#^*P* < 0.05 versus the WT/Ang II group.

Enhanced apoptosis contributed to Ang II-induced cardiac dysfunction. To elucidate the effect of Nlrp3 on Ang II-induced myocardial apoptosis, apoptosis in mouse heart was evaluated by TUNEL staining, and caspase 3 protein expression was analyzed by Western blotting. TUNEL staining revealed markedly more apoptotic cells in the WT/Ang II group (45.45 ± 2.03) than in the WT/vehicle control group (2.92 ± 0.21), and the number of TUNEL-positive cardiomyocytes induced by Ang II infusion was evidently inhibited by NLRP3 deletion (5.61 ± 0.32) (Figures 2(a) and 2(b)). These results were also confirmed by Western blotting. The protein levels of cleaved caspase 3 were significantly elevated (3.9-fold) in WT/Ang II mice (*P* < 0.05), and this increase was significantly attenuated in NLRP3^−/−^ mice (Figures 2(c) and 2(d)).

### 3.3. Echocardiography in Ang II-Induced Mice

**Figure 3:**
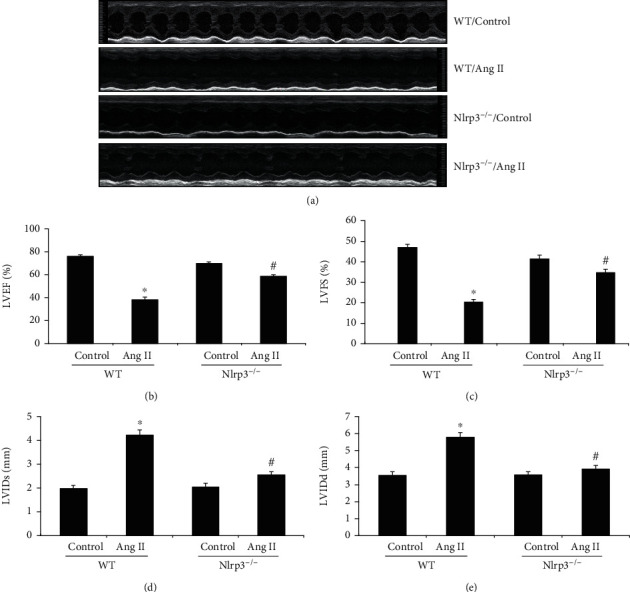
Representative M-mode left ventricle echocardiographic recording in Ang II-induced cardiomyopathy. (a) Representative M-mode left ventricle echocardiographic recording in Ang II-induced cardiomyopathy. (b) LVEF: left ventricular ejection fraction. (c) LVFS: left ventricular fractional shortening. (d) LVIDs: left ventricular internal dimension systole. (e) LVIDd: left-ventricular internal dimension diastole. All values are the means ± SEM (*n* = 6). ^∗^*P* < 0.05 versus the WT/vehicle control group; ^#^*P* < 0.05 versus the WT/Ang II group.

**Figure 4:**
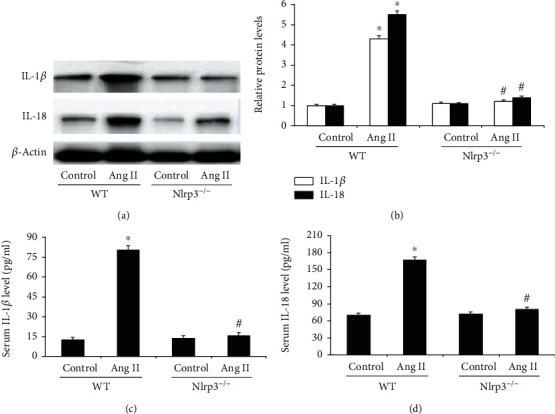
Nlrp3 deletion inhibited Nlrp3 inflammasome-related inflammatory cytokines. (a) The protein expression of heart IL-1*β* and IL-18 in the heart was determined by Western blotting and (b) the levels were normalized to *β*-actin in mice from the four groups. Serum IL-1*β* (c) and IL-18 (d) levels were measured via ELISA. All values are the means ± SEM (*n* = 6). ^∗^*P* < 0.05 versus the WT/vehicle control group; ^#^*P* < 0.05 versus the WT/Ang II group.

Cardiac dysfunction was detected by echocardiography, as shown in Figure 3. Compared with those of the WT/vehicle control group, the LVEF and LVFS in the WT/Ang II group were markedly decreased at week 4; Nlrp3 deletion significantly improved the LVEF and LVFS levels (Figure 3(b) and 4(c)). Similarly, the LVIDs and LVIDd were markedly increased in the WT/Ang II group in comparison with those in the WT/vehicle control group. However, Nlrp3 deletion significantly decreased the LVIDs and LVIDd (Figures 3(d) and 3(e)).

### 3.4. Nlrp3 Deletion Inhibited Nlrp3 Inflammasome-Related Inflammatory Cytokines

To examine the role of the Nlrp3 inflammasome and its downstream cytokines in Ang II-induced cardiomyopathy, the protein expression of IL-1*β* and IL-18 was evaluated by Western blotting. As shown in Figures 4(a) and 4(b), we observed that Ang II-induced Nlrp3 inflammasome activation and that heart IL-1*β* (4.3-fold) and IL-18 (5.5-fold) levels were significantly increased in the WT/Ang II group compared with the WT/vehicle control group. However, these effects were significantly inhibited in NLRP3^−/−^ mice. In addition, consistent with the Western blot results, we also demonstrated that serum IL-1*β* and IL-18 levels were upregulated in the WT/Ang II group, and these increased inflammatory cytokine levels were significantly attenuated by Nlrp3 deletion (Figures 4(c) and 4(d)).

### 3.5. Nlrp3 Deletion Attenuated Ang II-Induced Ultrastructural Morphology and Oxidative Stress

**Figure 5:**
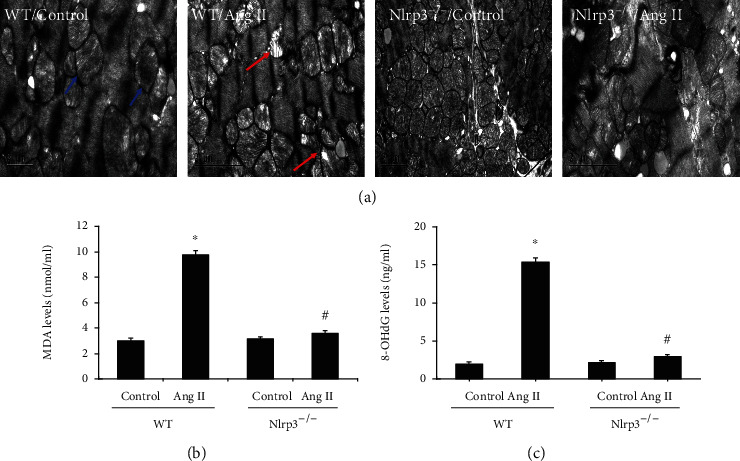
Nlrp3 deletion attenuated Ang II-induced ultrastructural morphology and oxidative stress. (a) Representative photomicrographs of changes in the ultrastructural morphology of mitochondria in mouse hearts (magnification ×12,000); the red arrow indicates swollen mitochondria; the blue arrow indicates the normal mitochondria. The MDA level (b) and serum 8-OHdG activity (c) were determined in the heart. All values are the means ± SEM (*n* = 6). ^∗^*P* < 0.05 versus the WT/vehicle control group; ^#^*P* < 0.05 versus the WT/Ang II group.

Observation of the ultrastructural alterations in mouse cardiomyocyte mitochondria demonstrated that in comparison with those of the WT/control group, the mitochondria in the WT/Ang II group were swollen with disorganized and fragmented cristae (Figure 5(a)). Nlrp3 deletion significantly ameliorated the morphological alterations in Ang II-induced cardiomyocytes. Moreover, because mitochondria are the main source of ROS, heart MDA levels and serum 8-OHdG activity were measured. The results showed that heart MDA and serum 8-OHdG levels were significantly increased in the WT/Ang II group. However, these oxidative stress markers were markedly reduced in the NLRP3^−/−^/Ang II group (Figures 5(b) and 5(c)).

### 3.6. Nlrp3 Deletion Ameliorated Ang II-Induced Mitochondrial Dysfunction in the Heart

**Figure 6:**
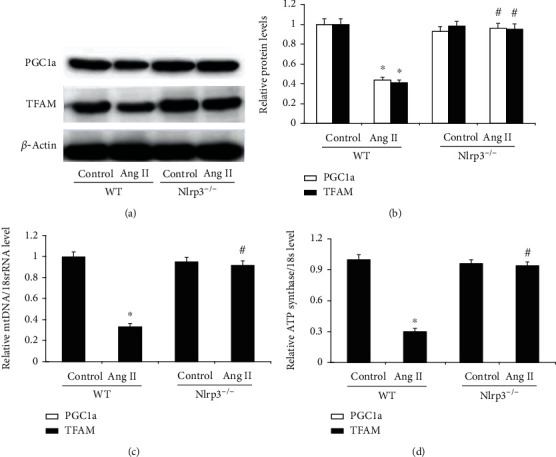
NLRP3 deletion ameliorates Ang II-induced mitochondrial dysfunction in the heart. (a) The protein expression of heart PGC1a and TFAM in the heart were measured by Western blotting and (b) the levels were normalized to *β*-actin in mice in the four groups. (c) Semiquantitative analysis of mitochondrial DNA (mtDNA) levels and (d) ATP synthase levels in mice from the four groups normalized to 18S and performed by real-time PCR. All values are the means ± SEM (*n* = 6). ^∗^*P* < 0.05 versus the WT/vehicle control group; ^#^*P* < 0.05 versus the WT/Ang II group.

Mitochondrial dysfunction is characterized by decreased mitochondrial biogenesis, disordered intracellular ATP synthesis, and mtDNA damage. In addition to alterations in mitochondrial morphology, the protein levels of PGC1a and TFAM in the heart were significantly decreased in the WT/Ang II group compared to those in the WT/vehicle control group (Figures 6(a) and 6(b)). Moreover, these effects were accompanied by significantly decreased mtDNA and ATP synthase levels in the WT/Ang II group compared with the WT/vehicle control group. The decreased protein levels of PGC1a and TFAM, mtDNA, and ATP synthase in the heart in the WT/Ang II group were significantly ameliorated in NLRP3^−/−^ mice at week 4 (Figures 6(c) and 6(d)).

## 4. Discussion

Inflammation plays an important role in various kinds of chronic diseases, including cardiomyopathy. The present study demonstrated that Nlrp3 inflammasome activation was involved in Ang II-induced cardiac fibrosis and hypertrophy, ultimately causing cardiomyopathy. Nlrp3 deficiency significantly attenuated cardiac inflammation, myocardial fibrosis and left ventricular dysfunction. In addition, the mitochondrial dysfunction resulting from Ang II infusion was alleviated in the Nlrp3-deficient group. This finding suggested that the Nlrp3 inflammasome caused mitochondrial dysfunction, which could be a potential therapeutic target to address Ang II-induced cardiac damage.

The RAS is an important cascade of essential regulators of various events in the cardiovascular system. Overactivation of Ang II, the strongest effector in the RAS, is involved in different types of cardiomyopathy [21]. The levels of Ang II have been shown to be elevated in both serum and cardiac tissue in a rat model with transverse aortic constriction. Ang II overaction directly causes cardiac remodeling, which is characterized by inflammation, fibrosis, myocyte apoptosis, and alterations in metabolism [22]. Recently, Ang II was also shown to be involved in the initial stage of inflammation and increased leukocyte recruitment through the increased expression of chemokines or inflammatory cytokines [23]. However, the potential molecular mechanism of inflammation-mediated cardiomyopathy in the Ang II-induced model remains unclear. In the present study, we found that long-term infusion of Ang II with a minipump was associated with cardiac inflammation and fibrosis, which led to heart failure. We did not notice any alterations in blood pressure in control mice with either the Nlrp3^+/+^ or Nlrp3^−/−^ genotype. Strikingly, Ang II-induced cardiac dysfunction was significantly alleviated in Nlrp3^−/−^ mice. These data indicate a pathogenic role of the Nlrp3 inflammasome in Ang II-induced cardiomyopathy.

The Nlrp3 inflammasome modulates innate immunity by activating caspase-1, which catalyzes the proteolytic processing and secretion of IL-1*β* and IL-18 [24]. Recently, the Nlrp3 inflammasome has been shown to mediate various types of diseases through the regulation of proinflammatory cytokines. Vilaysane et al. showed that chimeric mice harboring bone marrow-specific deletion of Nlrp3 were partly protected in a renal injury model [25]. Our previous study also showed that Nlrp3 deficiency attenuated renal fibrosis in a mouse unilateral ureteral obstruction model of CKD [14]. Recent studies have shown that NLRP3 inflammasomes recognize nonmicrobial danger signals and induce the release of the proinflammatory cytokine interleukin- (IL-) 1*β*, leading to sterile inflammation in cardiovascular disease. The Nlrp3 inflammasome has been shown to be involved in the development of cardiac contractile dysfunction in a CKD model [26]; the Nlrp3 inflammasome also plays a key role in myocardial fibroblasts after myocardial infarction [10]. Nlrp3 inflammasome activation and its downstream cytokines have been proposed as new biomarkers of cardiovascular risk.

Excess Ang II induces myocardial dysfunction. The mechanisms by which increased Ang II levels lead to fibrosis are multifactorial and likely involve fibroblast stimulation, ROS generation, inflammation, the upregulation of transcription factors such as nuclear factor kappa B, cytokines such as transforming growth factor beta and tumor necrosis factor alpha, and molecules such as plasminogen activator inhibitor-1 [27, 28]. However, the role of the Nlrp3 inflammasome in Ang II-induced cardiac fibroblasts is not completely understood. In the present study, we further examined Ang II-induced cardiomyopathy to identify the potential molecular mechanism. Our results demonstrated that cardiac fibrosis and myocardial apoptosis were increased in Ang II-induced mice. Moreover, left ventricular dysfunction, which was evaluated by echocardiography, was also significantly exacerbated in response to Ang II infusion, and genetic depletion of Nlrp3 markedly ameliorated cardiac function and fibrosis. Most importantly, Nlrp3^−/−^ mice were protected against Ang II-induced Nlrp3 inflammasome-related cytokine expression, and serum IL-1*β* and IL-18 and cardiac IL-1*β* and IL-18 protein levels were significantly decreased in the Ang II/Nlrp3 KO group. Previous studies showed that inflammation and oxidative stress are pivotal in the pathophysiology of cardiac remodeling [29, 30]. These results suggested that oxidative stress and mitochondrial dysfunction could activate the Nlrp3 inflammasome and induce cardiomyocyte damage. Furthermore, activation of the Nlrp3 inflammasome can exacerbate oxidative stress and mitochondrial dysfunction in cardiomyocytes. Therefore, complex crosstalk exists among Nlrp3 inflammasome activation, mitochondrial dysfunction, and oxidative stress. Taken together, these data indicate that inhibition of the Nlrp3 inflammasome may be a therapeutic strategy in Ang II-induced cardiovascular disease.

ROS plays a key role in the development of cardiac hypertrophy. NADPH oxidase is a transmembrane enzyme that generates superoxide (O_2_) by transferring an electron from NADPH to molecular oxygen [31]. A growing body of evidence has demonstrated that NADPH oxidase is the major source of Ang II-induced excessive ROS production in cardiomyocytes. Bendall et al. found that inactivation of the gp91phox subunit of NADPH oxidase significantly attenuated Ang II-induced hypertension and cardiac hypertrophy [32]. Moreover, the NADPH oxidase inhibitor, apocynin could decrease oxidative stress and cardiac fibrosis in Ang II-induced cardiac diastolic dysfunction [31]. However, recently, mitochondria have been recognized as another major source of ROS, which ultimately leads to cardiomyopathy including myocardial infarction and cardiac hypertrophy [33]. This notion is supported by recent research showing that Ang II markedly increased ROS production in mitochondria and that treatment with the mitochondrial-targeted antioxidant Mito-TEMPO significantly alleviated Ang II-induced cardiac hypertrophy [34]. These studies suggest that mitochondria are potential therapeutic targets for the treatment of cardiac hypertrophy and related diseases. Nemer also demonstrated that mitochondrial dysfunction was one of the earliest events in Ang II-induced cardiac failure, and mitochondrial dysfunction may provide new therapeutic opportunities for preventing the transition from compensated hypertrophy to heart failure [35].

However, the functions of the Nlrp3 inflammasome in mitochondria in Ang II-induced cardiac hypertrophy and cardiomyopathy remain unknown. A previous study proposed that Nlrp3 interacted with members of the cellular redox machinery, and other NLRs have been shown to target the mitochondrial matrix via an N-terminal-addressing sequence, mediating the JNK and NF-*κ*B pathways [36, 37]. Our previous work also showed that in the UUO model, Nlrp3 deficiency ameliorated renal function by attenuating mitochondrial dysfunction [38]. Furthermore, we also showed that mitochondrial ROS-mediated Nlrp3 inflammasome activation contributed to the development and progression of renal tubular injury [14, 38]. These data and previous research suggest that dual functions of Nlrp3 both upstream and downstream of mitochondrial dysfunction may exist in chronic kidney disease. However, the relationship between the Nlrp3 inflammasome and mitochondrial dysfunction in response to Ang II infusion in cardiomyocytes remains unclear. In the present study, we showed that mitochondrial function was markedly impaired in Ang II-induced cardiomyopathy, as evidenced by increased MDA and 8-OHdG levels, mitochondrial abnormalities, and decreased mtDNA copy numbers and ATP content. Nlrp3 depletion remarkably ameliorated Ang II-induced cardiac mitochondrial dysfunction. These data indicate the role of Ang II-induced Nlrp3 inflammasome-mediated mitochondrial dysfunction in the pathophysiological process of cardiomyopathy.

In conclusion, our study is the first to suggest that Nlrp3 inflammasome induced mitochondrial dysfunction is involved in Ang II-induced cardiomyopathy. Nlrp3 gene knockout significantly attenuated Ang II-induced cardiomyopathy by ameliorating mitochondrial dysfunction and reducing cardiac inflammation, oxidative stress, and fibrosis. Targeting the Nlrp3 inflammasome and/or mitochondria may be a therapeutic strategy in Ang II-induced cardiac diseases.

## Data Availability

The data that support the findings of this study are available from the corresponding author upon reasonable request.
